# Supplementary material for: EBNA1 SUMOylation by PIAS1 suppresses EBV lytic replication and enhances episome maintenance
Source: mBio. 2025 Dec 11;17(1):e02639-25. doi: 10.1128/mbio.02639-25 (PMC12802318; doi:10.1128/mbio.02639-25)
Supplement: Supplemental Figures — Figures S1 to S3. [file mbio.02639-25-s0001.docx]

**Supplemental FIgures**

**EBNA1 SUMOylation by PIAS1 Suppresses EBV Lytic Replication and Enhances Episome Maintenance**

Febri Gunawan Sugiokto^1,2 3^, Kun Zhang^4^, Yunash Maharjan^1,2,3^ and Renfeng Li^1,2,3^*

^1^Program in Microbiology and Immunology, University of Pittsburgh, Pittsburgh, PA 15219, USA

^2^Department of Microbiology and Molecular Genetics, University of Pittsburgh, Pittsburgh, PA 15219, USA

^3^Cancer Virology Program, Hillman Cancer Center, University of Pittsburgh Medical Center, Pittsburgh, PA 15232, USA

^4^Department of Oral and Craniofacial Molecular Biology, School of Dentistry, Virginia Commonwealth University, Richmond, Virginia, 23298, USA

*Corresponding author: renfeng.li@pitt.edu (RL)

**
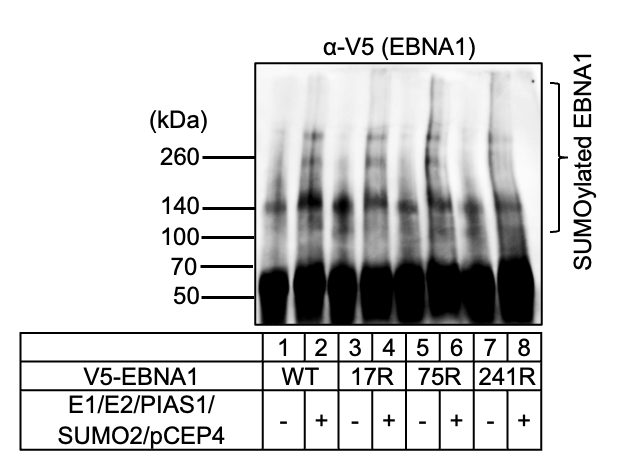
**

**Figure S1. The impact of K17R, K75R and K241R individual mutations on EBNA1 SUMOylation by PIAS1**.

Wild-type (WT) EBNA1 and single-site mutants (K17R, K75R, and K241R) were subjected to SUMOylation using E1, E2, SUMO2, PIAS1 and *oriP*-containing plasmid pCEP4. WB analysis was performed with anti-V5-HRP antibody. Bracket denotes poly-SUMOylated protein.


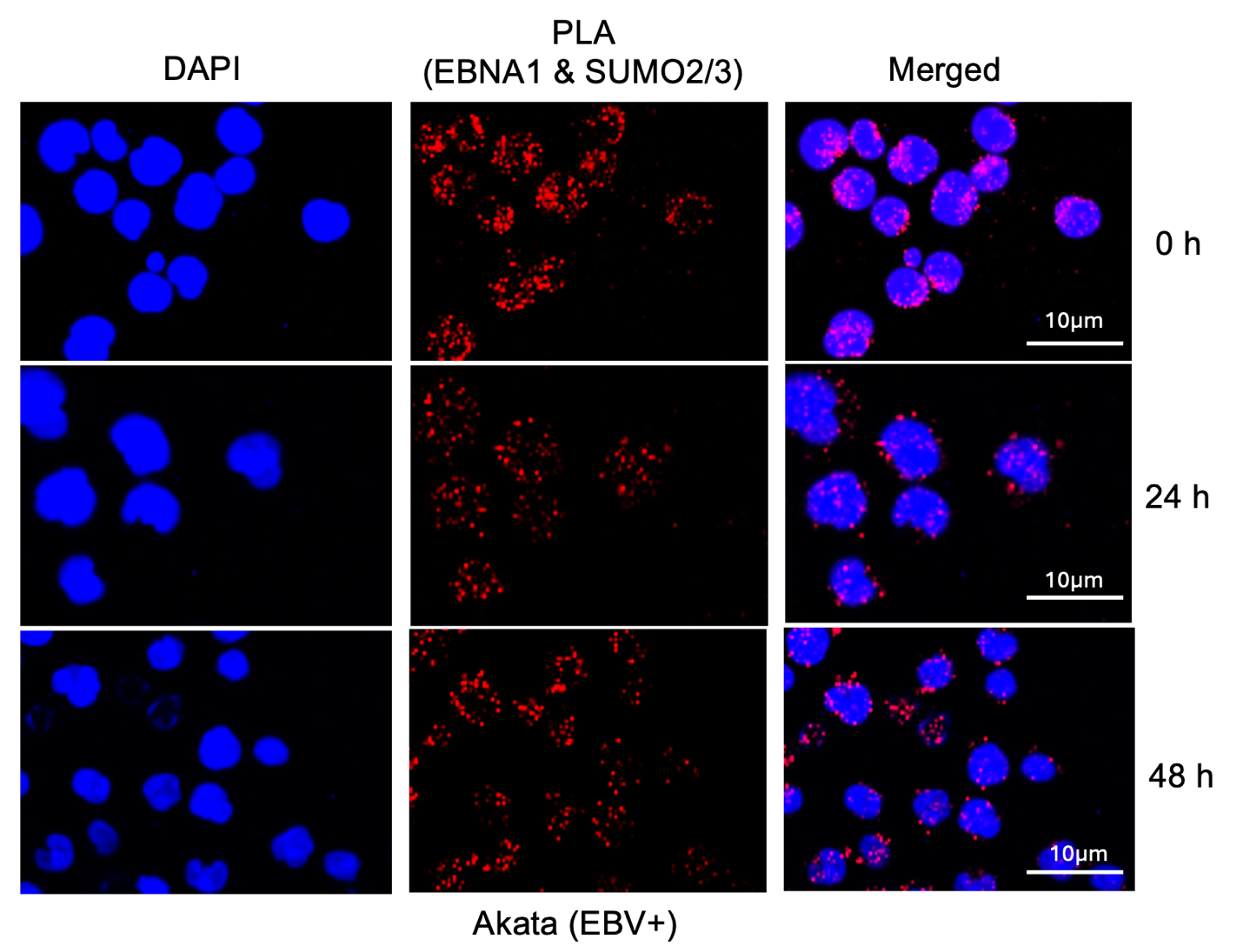


**Figure S2. Lytic reactivation reduces EBNA1 SUMOylation and promotes its translocation to the cytoplasm.**Akata (EBV+) cells were reactivated with anti-human IgG for the indicated 0, 24 and 48 hrs. The cells were then blocked with 3% BSA in PBS for 1 hour at room temperature, followed by incubation with mouse anti-EBNA1 and rabbit anti-SUMO2/3 antibodies. PLA probes were subsequently added for ligation and amplification. Nuclei were stained with DAPI and visualized using a Nikon AXR confocal microscope. The close proximity between EBNA1 and SUMO2/3 is indicated by red fluorescent PLA signals.


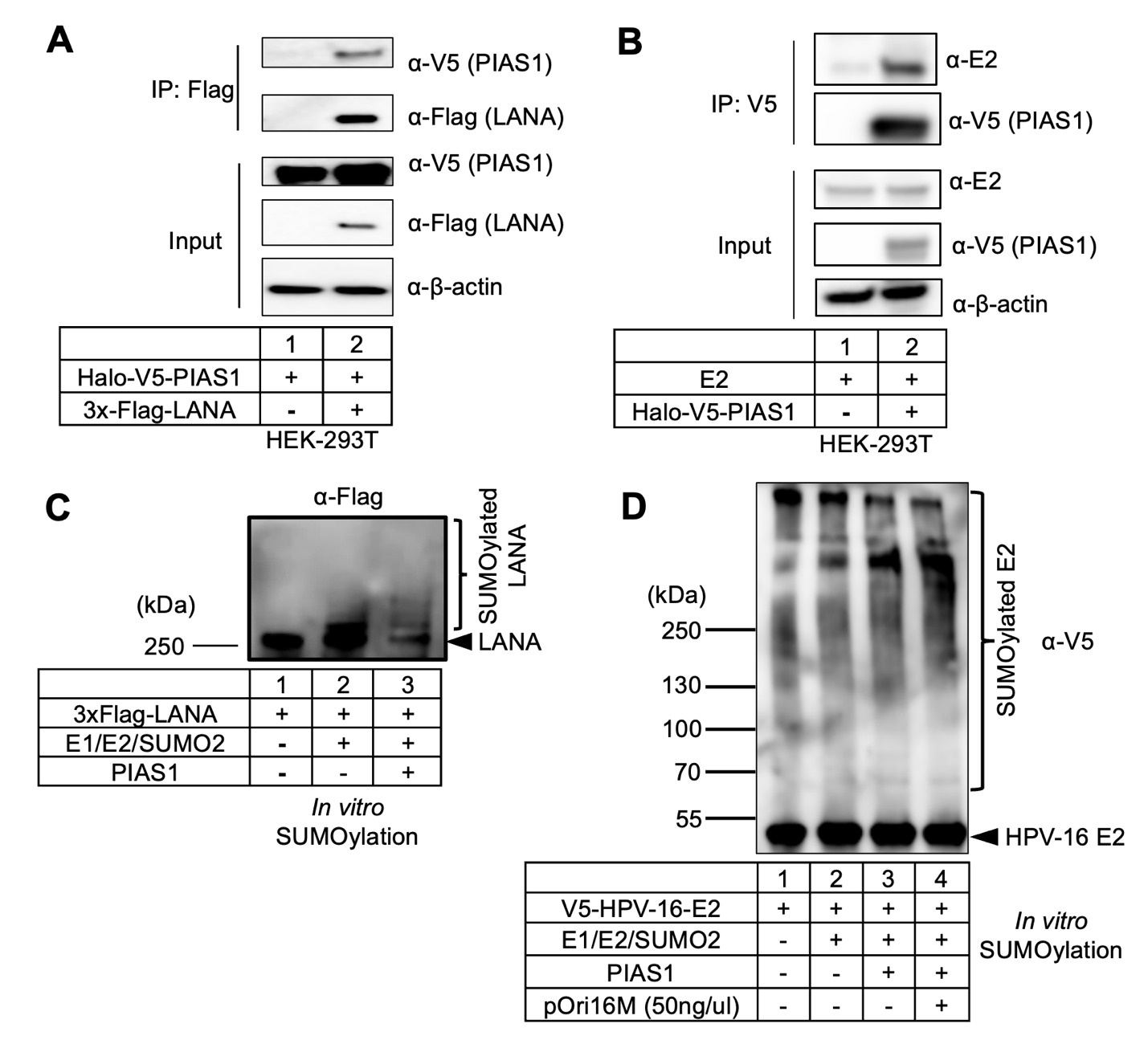


**Figure S3. PIAS1 promotes the SUMOylation of KSHV-LANA and HPV-16 E2.**

(**A**) HEK-293T cells were co-transfected with V5-PIAS1 and 3x-Flag-LANA plasmids. WB analysis showing that V5-PIAS1 is co-IPed with 3x-FLAG-LANA. Input: whole-cell lysates probed for V5-PIAS1, Flag-LANA, and β-actin (loading control). (**B**) HEK-293T cells were co-transfected with V5-PIAS1 and E2. WB analysis demonstrates that E2 is co-IPed with V5-PIAS1. Input: whole-cell lysates probed for V5-PIAS1, E2, and β-actin (loading control). (**C**) Precipitated 3x-FLAG LANA by anti-FLAG magnetic bead were subjected to *in vitro* SUMOylation reaction using E1, E2, SUMO2, and PIAS1. WB analysis was performed with anti-Flag-HRP antibody. Bracket denotes SUMOylated LANA. (**D**) Purified V5-E2 were subjected to SUMOylation using E1, E2, SUMO2, PIAS1, and HPV-16 *oriP*-containing plasmid, pOri16M. WB analysis was performed with anti-V5-HRP antibody. Bracket denotes SUMOylated E2.
